# *Euonymus alatus* Leaf Extract Attenuates Effects of Aging on Oxidative Stress, Neuroinflammation, and Cognitive Impairment

**DOI:** 10.3390/antiox13040433

**Published:** 2024-04-02

**Authors:** Pallavi Gurung, Junmo Lim, Til Bahadur Thapa Magar, Rajeev Shrestha, Yong-Wan Kim

**Affiliations:** Dongsung Cancer Center, Dongsung Pharmaceuticals Corporation, Daegu 41061, Republic of Korea; gp20@ds-pharm.co.kr (P.G.); ijm15@ds-pharm.co.kr (J.L.); til09@ds-pharm.co.kr (T.B.T.M.); shrestha.144@osu.edu (R.S.)

**Keywords:** aging, euonymus alatus leaf extract, neuroinflammation, cognitive impairment, oxidative stress

## Abstract

Our study aimed to explore the impact and mechanism of *Euonymus alatus* leaf extract on age-dependent oxidative stress, neuroinflammation, and progressive memory impairments in aged mice. Twenty-four-month-old mice received EA-L3 (300 mg/kg/day) or the reference drug, donepezil (DPZ, 5 mg/kg/day), for 6 weeks, and learning and memory functions were detected using the Passive Avoidance Test (PAT). As expected, cognitive function deficits were detected in aged mice compared with young mice, and these deficits were significantly mitigated by dietary treatments with EA-L3. In parallel, it upregulated the brain-derived neurotrophic factor (BDNF) and subsequently activated the extracellular-signal-regulated kinase (ERK)/cAMP response element-binding (CREB) signaling in the mouse hippocampus and scopolamine-induced B35 and SH-SY5Y neuroblastoma cells. EA-L3 showed strong anti-inflammatory effects with decreased NF-κBp65, cyclooxygenase 2 (COX-2), and tumor necrosis factor alpha (TNF-α), increased interleukin (IL)-10, and doublecortin (DCX) protein expression in the hippocampus of aged mice. Similar results were also confirmed in LPS-induced BV-2 microglia and neuroblastoma cells upon treatment with EA-L3 extract. In addition, EA-L3 notably dose-dependently decreased ROS in BV2 cells after exposure to LPS. Taken together, EA-L3 might be used as a dietary supplement to alleviate oxidative stress, the deterioration of hippocampal-based memory tasks, and neuroinflammation in elderly people.

## 1. Introduction

Aging results in progressive physiological changes that contribute to the loss of both biological and mental function [1,2]. Age-related changes influence the gradual decline of the immune system. The balance between pro- and anti-inflammatory cytokines is mostly maintained in the brain, but as people age, the intrinsic dysfunction of the immune system, known as “immunosenescence”, shifts the balance in favor of the pro-inflammatory state [3,4]. This leads to a low-level chronic inflammatory state in the absence of neurologic disease [5], which increases the susceptibility to conditions like neurodegenerative diseases such as Alzheimer’s disease (AD) and Parkinson’s disease (PD) [6]. Another culprit that drives pro-inflammatory conditions during aging might be due to changes in gene expression [7,8,9]. The impaired cognitive performance in the elderly is associated with chronic systemic inflammation, which impairs neurogenesis and promotes neurodegenerative diseases [10]. In this context, a deeper understanding of the aging process has the potential to prolong a healthy life and prevent aging-related diseases [11,12].

Naive T cells decrease with age, which causes T cell generation to decline. This, in turn, causes the synthesis of anti-inflammatory hormones to increase, which speeds up the proinflammatory profile [13,14]. In addition, as people age, NK cell activity and gut flora levels decline, boosting the activation of innate immunity. [15]. Such an augmented neuroinflammatory cytokine response in the aged brain reduces neuronal synaptic plasticity and worsens neurobehavioral functions [5]. It is well recognized that the normal aging brain maintains activated and primed microglia [3]. Even in the absence of detectable disease, the glia population underwent age-related changes like the enlargement or development of phagocytic microglia [16], increased HLA-DR expression on the microglia surface [17], and increased sensitivity of the brain’s environment. Excessive levels of ROS can harm neurons and impair cognitive function, which can result in age-related diseases that encourage oxidative stress [18]. In addition, oxidative stress and higher levels of proinflammatory cytokines like IL-6 or IL-1β are found to be the main causes of the abundance of reactive glia in the aged brain [5,19].

CREB is a cellular transcription factor that regulates the expression of essential genes in dopaminergic neurons and plays a role in long-term memory formation and neuronal plasticity [20]. It has been known that CREB, a pro-youthful factor from young blood, can repair age-related cognitive deficits [21]. According to Yu et al., aged brains have lower CREB levels and activity. Such increased CREB activity helps to improve age-related impairments [22]. Neurogenesis occurs in the dentate gyrus (DG) via the mitosis of neural progenitor cells located in the sub granular zone (SGZ) and may play an important role in the maintenance of hippocampal functions such as learning and memory. With age, neurogenesis, a process of the formation of new neurons from neural stem cells and progenitor cells, is known to decrease. Clinical and animal studies have reported hippocampal volume decreases during aging, which are associated with reductions in the branching pattern of dendrites or in the densities of fibers projecting into the hippocampus along with reduced neurogenesis, and this reduction contributes to impaired cognition in the aging brain [22,23,24,25,26].

The majority of current drug treatments are now being developed to combat the neuroinflammation that increases with age [27]. However, only few drugs are approved due to their side effects, which includes nephrotoxicity, hepatotoxicity, nausea, etc. Much of today’s pharmaceutical industry uses drug substances that are obtained from natural sources. Additionally, plant sources have fewer adverse effects, while plant-derived agents have anti-neuroinflammatory, neurogenic, and cognitive enhancing effects [28]. Therefore, herbal medicines might be a better option for treating aging or aging-associated diseases. Likewise, *E. alatus* is known to have antioxidant and neuroprotective effects against inflammation, cognitive-impairment, and AD [29,30,31]. Major components of *E. alatus* were identified as sesquiterpenes, sesquiterpene alkaloids, triterpenes, flavonoids, and phenolic compounds [32].

In the present study, we clarified the anti-inflammatory and cognitive-improving effects as well as the underlying mechanism of the actions of EA leaf extract in an aged mouse model. We employed in vitro cell models, such as lipopolysaccharide (LPS) or scopolamine-treated neuroblastoma (SH-SY5Y and B35) and LPS-induced murine microglial BV2 cells, to study the anti-neuroinflammatory or anti-cognitive effects of EA-L3 extract.

## 2. Materials and Methods

### 2.1. Chemicals

LPS from *Escherichia coli* (0111:B4), donepezil, and scopolamine hydrobromide were purchased from Sigma Chemical Co. (St. Louis, MO, USA). DMEM, Fetal Bovine serum, Antimycin A, and TryPLE express were acquired from Gibco (ThermoFisher Scientific, Waltham, MA, USA). Donepezil was obtained from Sigma-Aldrich Chemical Co., St. Louis, MO, USA. The primary antibody against BDNF (bsm-52368R) was obtained from Bioss (Beijing, China), whereas antibodies against p-p65NF-κB (CST3031), p65NF-κB (CST3034), p-ERK (CST9102), ERK (CST9102), p-CREB (CST9198), and CREB (CST4820) were procured from Cell Signaling Technology Inc. (Beverly, MA, USA). Antibody against TNF-α (52B83) was sourced from Novus Biologicals, Inc. (Centennial, CO, USA), IL-10 from Abbiotec, Inc. (Escondido, CA, USA), and COX-2 (Sc19999) from Santa Cruz Biotechnology, Inc. (Santa Cruz, CA, USA), respectively. Anti-Doublecortin (ab 18723) and anti-β-Actin (ab8226) antibodies were purchased from Abcam (Cambridge, MA, USA).

### 2.2. Preparation of the Euonymus alatus Leaves Extract and Drug Preparation

**Figure 1:**
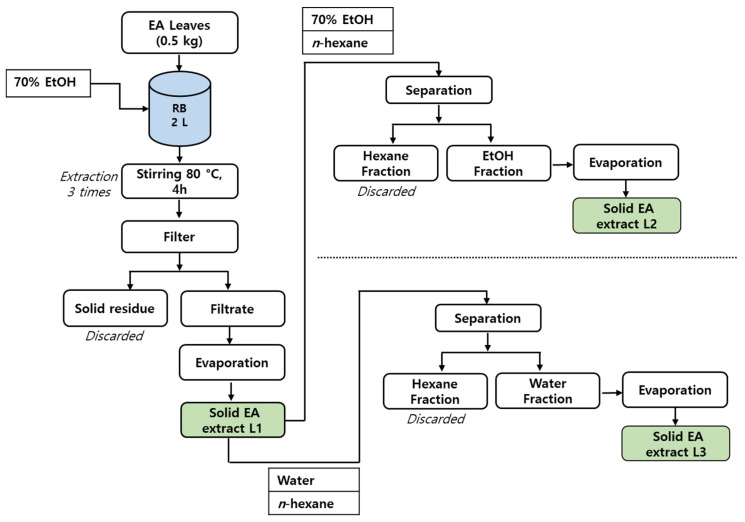
Schematic representation of EA leaves extract L1, L2, and L3 preparation.

The leaves of voucher specimen (1643205123) were collected from Asan, Republic of Korea, in August 2021. EA was identified by Biodiversity Research Institute of Jeju Technopark (Jeju 63608, Republic of Korea) and was deposited in *Euonymus alatus* (Thunb.) Siebold (Plantae > Magnoliophyta > Magnoliopsida > Celastrales > Celastraceae > *Euonymus* L., Sp. Pl) (Herbarium no. 20100718005). The EA plants were air-dried and stored at −20 °C before using for extraction. Three types of ethanol extracts: EA leaf extract L1, extract L2, and extract L3 were prepared as shown in schematic representation (Figure 1). The dried EA leaves (0.5 kg) were extracted three times with 70% ethanol (3 × 5 L) for 4 h at 80 °C. The mixture was cooled to room temperature and filtered. The filtrate was evaporated under reduced pressure to yield the concentrated leaves extract L1 (56 g). Then, leaf extract L1 (10 g) was dissolved in 70% ethanol and fractionated with hexanes (3 times). A portion of 70% ethanol was collected and evaporated under reduced pressure followed by drying in a vacuum oven for two hours (60 °C) to yield the leaf extract L2 (8 g). Similarly, leaf extract L1 (10 g) was dissolved in water and hexane (1:1 *v*/*v*), followed by liquid–liquid separation (3 times). A portion of water was collected and evaporated under reduced pressure followed by vacuum drying for 2 h at 60 °C to yield leaf extract L3 (7.5 g). Later, leaf extracts L1–L3 were stored at 4 °C before further analysis.

### 2.3. HPLC/MS Methods

Waters Alliance HPLC system (Waters, Houston, TX, USA), consisting of a binary pump, an online degasser, and a diode array detector (DAD), was used to analyze the chromatograms of leaf extracts. A Sunfire C18 (4.6 mm × 250 mm, 5 µm) column was used at room temperature. The mobile phase consisted of 0.08% TFA water (A) and acetonitrile (B) using a gradient program of 5–15% (B) in 0–5 min, 15–35% (B) in 5–40 min, 35–50% (B) in 40–55 min, 50–65% (B) in 55–60 min, 65–100% (B) in 60–70 min, and 100% (B) in 70–80 min. All the samples were filtered through 0.22 μm syringe filter prior to HPLC analysis and the chromatograms were monitored at 360 nm. Mass spectra were recorded using Waters micromass ZQ equipped with electrospray ionization (ESI) under positive ionization mode.

### 2.4. Cell Cultures

B35, a rat neuroblastoma cell line, was provided from the American Type Culture (ATCC, CRL-2754). Human neuroblastoma SH-SY5Y cells were obtained from the Korean Cell Line Bank (KCLB 22266, Seoul, Republic of Korea). Cells were maintained in Dulbecco’s Modified Eagle’s Medium (DMEM) with 10% fetal bovine serum and 1% antimycin A at 37 °C in a humidified atmosphere under 95% air and 5% CO_2_.

RAW264.7 cells were procured from the Korean Cell Line Bank (KCLB 40071, Seoul, Republic of Korea) and maintained in RPMI-1640 full medium supplemented with 10% fetal bovine serum and 1% antimycin A. The murine BV2 microglial cell lines were obtained from the KCLB, Seoul, Korea, and were cultured in DMEM containing 10% fetal bovine serum and 1% antimycin A. The cells were maintained in a humid atmosphere of 5% CO_2_ at 37 °C.

### 2.5. Estimation of Cell Viability

RAW264.7 cells were plated overnight in the 96-well plates at a density of 2 × 10^5^ cells per well, while BV2, B35, and SH-SY5Y cells were seeded at a density of 5 × 10^4^ cells per well. The cells were then treated with EA-L1, EA-L2, and EA-L3 extracts at concentrations of 3, 5, 10, 30, 50, 100, 300, 500, and 1000 μg/mL. DPZ (3–200 μM), a standard drug, was also treated with respective cell lines to determine the cell viability. MTT (0.2 mg/mL) was added to cultures, followed by incubation at 37 °C for 3 h. Then the supernatant was aspirated, and 100 μL DMSO was added to dissolve the formazan. After 30 min of shaking, the absorbance at 540 nm was measured using a microplate reader. Data are expressed as percentage of cell viability compared to the control.

### 2.6. Intracellular ROS Measurement

Intracellular ROS accumulation was detected using 2′-7′ dichlorofluorescein diacetate (DCF-DA, a fluorescent dye). BV2 cells were treated with LPS (1 μg/mL) in the presence or absence of EA-L3 (5–300 μg/mL). After 24 h incubation, cells were washed twice with DPBS. Cells were stimulated with DPBS containing 5 μM DCF-DA for 30 min at 37 °C. After removal of DCFDA, cells were washed with buffer, and intracellular ROS levels were measured using a SparkMultimode microplate reader (Tecan Trading AG, Männedorf, Switzerland).

### 2.7. Animal

Young (6 weeks) and aged (24 months) male C57BL/6 mice were purchased from Orient Bio (Seongnam, Republic of Korea). The animals were housed and fed in a specific pathogen-free animal facility at the Dongsung Cancer Center, Daegu, for 7 days. The experiments were performed according to the guidelines of the Institutional Animal Care and Use Committee of the Dongsung Cancer Center under protocol IACUC # ds0022010107-2. The experiments were carried out in compliance with the ARRIVE guidelines.

#### 2.7.1. Experimental Groups

Young C57BL/6 mice (*n* = 5) were selected as Group 1. Aged C57BL/6 mice (*n* = 15) were randomly divided into three subgroups with 5 mice in each group: Group 2 (vehicle-treated aged mice), Group 3 (aged mice fed with EA-L3), and Group 4 (aged mice fed with DPZ). The drugs were prepared in 0.9% physiological saline as a diluent. Animals received saline, EA-L3 extract (300 mg/kg), and DPZ (5 mg/kg) once daily in groups 2, 3, and 4, respectively, orally. The testing was performed over a period of 42 days, up to the the end of the experimental period.

#### 2.7.2. Passive Avoidance Test

Mouse behavioral testing was performed on days 40 and 41. The PAT was conducted in equally placed illuminated and non-illuminated compartments (Jeong do BNP, Seoul, Republic of Korea), separated by a small door. A habituation test was conducted to familiarize the animals with the device. Individual animals were placed in the apparatus and given 60 s to freely explore the non-illuminated box until the light was turned on and the door was unlocked. The mice were allowed to enter the dark component and were removed. The initial latency time of the mice entering the dark room after opening the door was timed. A training session was performed on day 40. Each mouse was placed in a lighted compartment with the door closed for 10 s. When the door was opened, the mouse was allowed to enter into the dark room. After the mice stepped down on the grid floor with all four legs and the tail, the door was closed, and an electric shock to the feet (0.5 mA, 3 s) was applied. The mouse was returned to the cage after 10 s. After 24 h, during the testing phase, the animals were placed back into the lighted box, and the latency to enter the dark box was determined to measure memory retention (up to 300 s). Mice were given a score of 300 s if they failed to enter in this time frame.

### 2.8. Western Blot Analysis

B35 or SH-SY5Y cells were seeded at the density of 4 × 10^5^ cells/well and pre-treated with or without EA-L3 (50 and 100 µg/mL) and DPZ (5 µM), respectively. Then the cells were treated for 24 h with scopolamine (2 mM).

B35 or SH-SY5Y cells were seeded at the density of 4 × 10^5^ cells/well and pre-treated with or without EA-L3 (10, 30, 50 and 100 µg/mL) and DPZ (5 µM), respectively. Then the cells were treated for 24 h with LPS (1 μg/mL).

After administering the medication for 1 h, the mice were sacrificed by cervical dislocation. To isolate the bilateral hippocampus areas, the brain was removed and kept at −80 °C in preparation for Western blot analysis.

The cells and hippocampal tissues were then lysed with RIPA buffer containing 0.15 M Sodium chloride, 1% Triton X-100, 1% Sodium deoxycholate, 0.1% SDS, and 50 mM Tris, which was adjusted to pH 7.5. The protein content in the supernatant was quantified with Bio-Rad, protein assay reagent, and the cell lysates were boiled in sodium dodecyl sulfate (SDS) sample buffer at 95–100 °C for 5 min and then analyzed on SDS–PAGE. Primary antibodies against BDNF, p-ERK, ERK, p-CREB, CREB, TNF-α, IL-10, DCX, and COX-2 were diluted 1:1000 in 5% skim milk/Tris-buffered saline with Tween 20 detergent buffer (TBST). Horseradish peroxidase-conjugated immunoglobulin G (Cell Signaling Technology, Danvers, MA, USA) was used as the secondary antibody. Immunoreactive bands were identified using the ECL chemiluminescent reagent (Pierce Biotechnology, Rockford, IL, USA).

### 2.9. Stastical Analysis

The obtained data were expressed as mean ± standard deviation (SD) and were analyzed by one-way analysis of variance (ANOVA) with Tukey’s post hoc test, using GraphPad Prism software (version 5.01, Inc., 2007, San Diego, CA, USA). Probability values less than 0.05 were considered statistically significant.

## 3. Results

### 3.1. HPLC/MS of Leaves Extract

**Figure 2:**
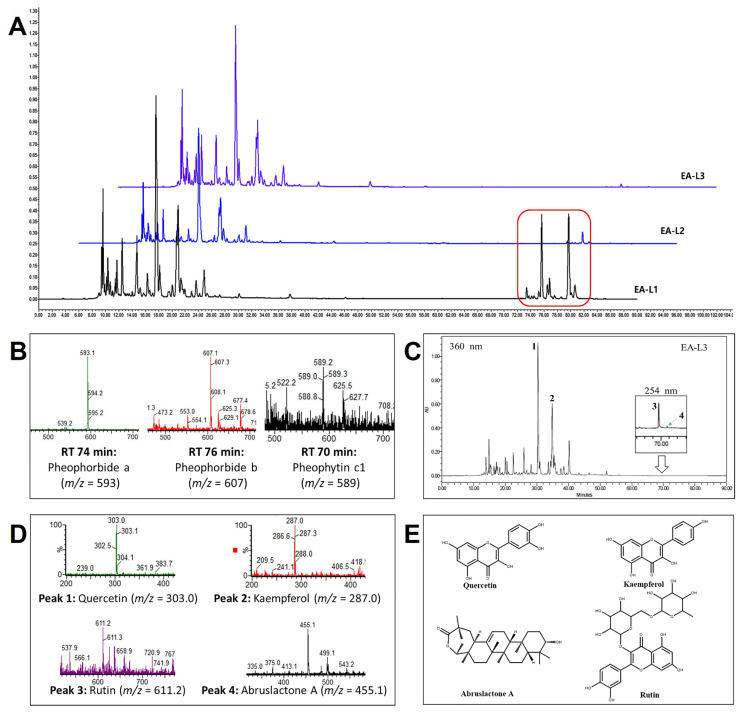
HPLC/MS analysis of EA leaf extracts. (**A**) Comparison of HPLC chromatogram of EA-L1, EA-L2, and EA-L3 extracts. Red rectangle represents the chlorophyll-related hydrophobic compounds present in EA-L1. (**B**) Chlorophyll-related compounds identified by HPLC/MS analysis of EA L1 extract. (**C**) HPLC chromatogram of EA-L3 at 360 nm and 254 nm (for triterpenes). (**D**) Corresponding molecular ion peaks of identified flavonoids and triterpene on EA-L3. (**E**) Chemical structure of compounds (Peak 1: Quercetin, Peak 2: Kaempferol, Peak 3: Rutin, and Peak 4: Abruslactone).

To study the comparison of components in the leaf extracts, the leaf extracts were analyzed through HPLC (Figure 2A). The major bioactive components were observed to have a shorter retention time (RT). In the case of EA leaf extract L1, chlorophyll-related compounds were detected in 70–80 min of RT (Figure 2A). In particular, the HPLC/MS analysis of EA-L1 displayed the presence of pheophytin c1, pheophorbide a, and b at 70, 74, and 76 min RT, respectively (Figure 2B). However, chlorophyll-related non-polar hydrophobic compounds were not found in L2 and L3 due to the washing with hexane during the preparation. Three major types of flavonoids, namely quercetin (peak 1), kaempferol (peak 2), and rutin (peak 3) were identified in EA-L3 at 360 nm (Figure 2C–E). The extraction of the HPLC chromatogram at 254 nm revealed the presence of triterpenes such as abruslactone A (peak 4).

### 3.2. Effect of EA-Leaves Extracts on Different Cell Viabilities

**Figure 3:**
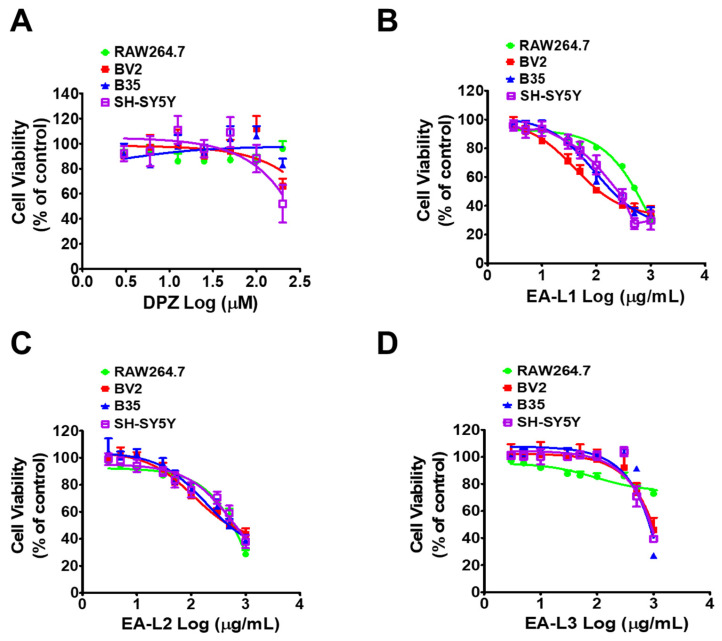
IC50 values for DPZ, EA-L1, EA-L2, and EA-L3 in cytotoxicity assays using RAW264.7 BV2, B35, and SH-SY5Y cells. Cell lines were treated with a range of drug concentrations as indicated to test the cytotoxicity of DPZ, EA-L1, EA-L2, and EA-L3. Cell lines were treated with the drug for 24 h. MTT assay was performed, and absorbance was measured by determining the absorbance at 490 nm. A cytotoxicity assay was studied for (**A**) DPZ, (**B**) EA-L1, (**C**) EA-L2, and, (**D**) EA-L3, respectively, in RAW264.7 BV2, B35, and SH-SY5Y cell lines. IC50 values were determined using Graph Pad Prism 5 software.

Initially, the cytotoxicity of each extract was tested on RAW 264.7 cells, a mouse macrophage, BV2 microglial, B35, a rat neuroblastoma, and SH-SY5Y, a human neuroblastoma cell, to establish the non-cytotoxic doses of EA-L1, L2, and L3. RAW264.7 and BV2 cells were utilized to evaluate the systemic effect, as macrophages are present in all tissues and are the standard cell model to assess the anti-inflammatory activity promoted by plant extracts. We also determined the cytotoxicity of the standard drug DPZ in the respective cell lines. All four cell lines were treated with DPZ (3–200 μΜ) for 24 h. DPZ showed no toxicity to the RAW264.7 and B35 cell lines up to 200 μΜ (IC50 > 200 μM). However, DPZ showed slight toxicity at 200 μΜ in BV2 and SH-SY5Y (IC50 > 200 μM) (Figure 3A).

RAW264.7, BV2, B35, and SH-SY5Y cells were exposed to different doses of the EA-L1, L2, and L3 extracts (3–1000 μg/mL) for 24 h to evaluate the effect of the extracts on cell viability by an MTT colorimetric assay (Figure 3B–D). Among the EA leaf extracts tested, EA-L1 showed the greatest cytotoxicity in all the above-mentioned cell lines. The IC50 values of EA-L1 were 112.6, 117.2, 202, and 227.3 μg/mL in RAW264.7, BV2, B35, and SH-SY5Y cells, respectively, after 24 h (Figure 3B). EA-L2 showed a lesser cytotoxicity than EA-L1, but a higher cytotoxicity than EA-L3, with IC50 values of 579.4, 535.9, 495.7, and 651.2 μg/mL in RAW264.7, BV2, B35, and SH-SY5Y cells, respectively, after 24 h (Figure 3C). However, even at the highest concentration tested, EA-L3 was not cytotoxic to RAW264.7 (IC50 < 1000 µg/mL) or BV2 (IC50: 961.8 μg/mL) at concentrations up to 500 μg/mL, and B35 up to 300 μg/mL (IC50: 822.8 μg/mL), while SH-SH-SY5Y cells exhibited an IC50 of 889.6 μg/mL (Figure 3D). Lower cytotoxicity must be due to the absence of non-polar chlorophyll-related hydrophobic compounds in EA-L3, as shown in the HPLC/MS readings. Hence, in light of these findings, EA-L3 was selected for further study.

### 3.3. Effect of EA-L3 in Modulation of CREB/BDNF Signaling in Scopolamine-Induced Neuroblastoma Cells

**Figure 4:**
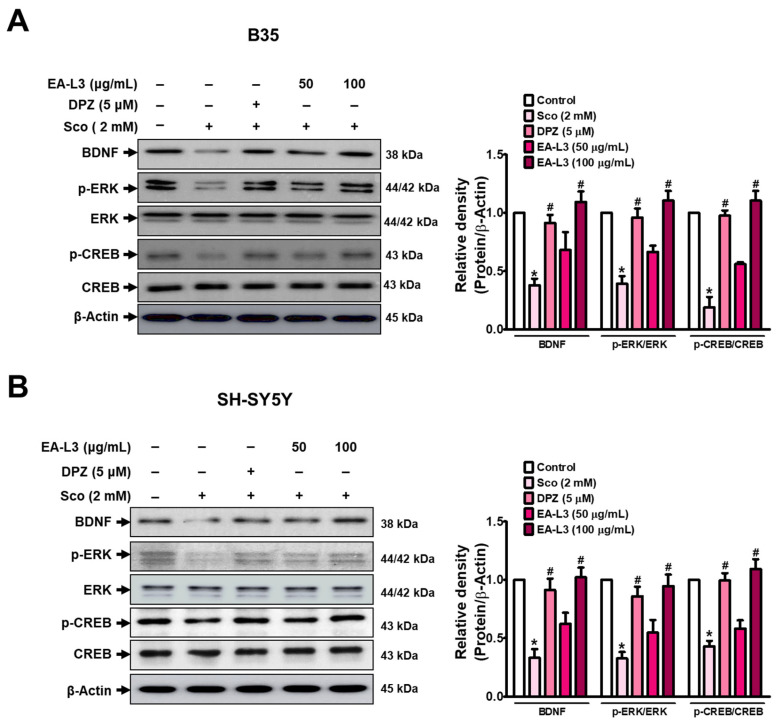
Effect of EA-L3 on scopolamine-triggered expression of memory-related molecular markers in B35 and SH-SY5Y cells. Cells were treated with EA-L3 at the indicated concentration, and the extracted protein was analyzed to determine BDNF, p-ERK, and p-CREB protein levels in (**A**) B35 and (**B**) SH-SY5Y cells. The data presented are the means ± SD of three independent experiments and were analyzed by one-way ANOVA with Tukey’s post hoc test. * *p* < 0.05 vs. control group; # *p* < 0.05 vs. scopolamine-treated group.

BDNF is crucial for the growth, survival, and differentiation of neurons as the central nervous system develops. BDNF exerts its neuroprotective effects mainly by activating the BDNF/CREB signaling pathway. The pathway controls several neuronal processes and is important for maintaining normal brain function [33,34]. In accordance, we assessed how EA-L3 modulated these neuronal enrichment factors in the scopolamine-treated neuroblastoma cell lines B35 and SH-SY5Y, respectively (Figure 4A,B and Figure S2). In our earlier study, we had found that scopolamine at a concentration of 2 mM significantly decreased the viability of B35 cells [31]. In the present study, scopolamine decreased the expression levels of p-CREB, p-ERK, and BDNF in the B35 cell line, but treatment with EA-L3 significantly upregulated the expression levels of p-CREB, p-ERK, and BDNF protein, similar to the DPZ-treated groups. At a higher dose, EA-L3 (100 μg/mL) restored the p-CREB, p-ERK, and BDNF expressions to a greater extent than the DPZ-treated and scopolamine-treated groups. SH-SY5Y cells also showed a similar response to EA-L3 treatment, with gradually decreasing p-CREB, p-ERK, and BDNF expression levels at 50 and 100 μg/mL. The results suggest that EA-L3 extract restored the scopolamine-induced downregulation of BDNF, p-ERK, and p-CREB.

### 3.4. Effect of EA-L3 on LPS-Induced ROS Generation and Expression Levels of Inflammatory Markers in BV2 Microglial Cells

**Figure 5:**
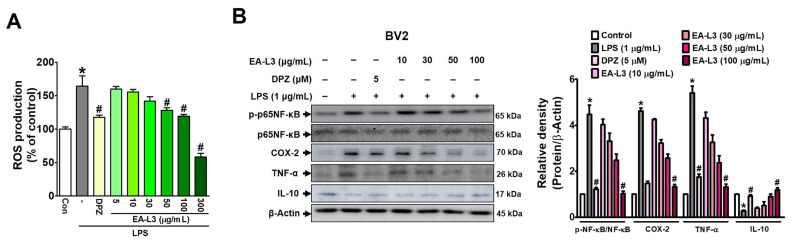
Effect of EA-L3 on inhibiting ROS production and inflammatory markers in LPS-induced BV2 microglial cells. (**A**) BV2 cells were pre-treated with DPZ (5 μΜ) or EA-L3 (0–300 μg/mL) for 3 h, followed by exposure to 1 μg/mL of LPS for 24 h. The cells were then loaded with 5 μM 2′-7′dichlorofluorescin diacetate (DCFH-DA) and fluorescence intensities were measured using a microplate reader. The data presented are the means ± SD of three independent experiments. * *p* < 0.05 vs. control group; # *p* < 0.05 vs. LPS-treated group. (**B**) The cell lines were treated alone or co-treated with LPS (1 μg/mL) and DPZ (5 μΜ) or EA-L3 (10–100 μg/mL) for 24 h and total protein was extracted. A Western blot was performed for the analysis of p-p65NF-κB, COX-2, TNF-α, and IL-10 protein levels, respectively. The data presented are the means ± SD of three independent experiments and were analyzed by one-way ANOVA with Tukey’s post hoc test.* *p* < 0.05 vs. control group; # *p* < 0.05 vs. LPS-treated group.

The study next investigated whether LPS-induced ROS levels were indeed decreased in BV-2 microglial cells with EA-L3 (≥300 μg/mL) (Figure 5A). We chose an EA-L3 concentration of up to 300 μg/mL for this experiment since it did not affect cell viability. These results confirmed that EA-L3 dramatically suppressed intracellular ROS generation in a concentration-dependent manner compared to the vehicle-treated control. DPZ (5 μM) significantly blocked LPS-mediated ROS production in BV2 microglial cells.

As evident, inflammatory cytokines such as TNF-α and IL-6 are important markers of activated microglial cells during neuroinflammation [18]. On the other hand, IL-10 is an anti-inflammatory cytokine that controls inflammatory processes [35]. To evaluate the role of EA-L3 in LPS-induced inflammatory responses, we examined the expressions of p-p65NF-κB, TNF-α, COX-2, and IL-10 (anti-inflammatory cytokines) in microglial BV2 cells (Figure 5B and Figure S3). Interestingly, we identified the activation of NF-κB, a key component of the inflammatory response, in activated microglia. Our results demonstrate that EA-L3 treatment significantly reduces the levels of the LPS-induced phosphorylation of p65NF-κB expression. Interestingly, our study shows that the upregulated expression of TNF-α was reduced by pre-treatment with EA-L3 in a concentration-dependent manner in comparison to the vehicle-treated control. Additionally, COX-2 plays an important role in the inflammatory cascade and its expression causes secondary damage to neurons [36]. Cells treated with EA-L3 (100 μg/mL) showed a significant reduction in p65NF-κB, COX-2, and TNF-α, similar to cells treated with DPZ (5 μΜ). Our findings indicate that the increased protein expression of COX-2 in BV-2 cells due to LPS-induction was reduced by EA-L3 in a dose-dependent manner. In contrast, the LPS-downregulated expression of IL-10 was enhanced by EA-L3 dose-dependently compared to the vehicle-treated control. Cells treated with EA-L3 (100 μg/mL) showed a significant increase in IL-10 expression, similar to DPZ treatment. Therefore, our data suggest that EA-L3 plays an important role in inhibiting the production of inflammatory mediators and microglial activation.

### 3.5. Effect of EA-L3 on LPS-Induced Proinflammatory and Anti-Inflammatory Mediators in Neuroblastoma Cells

**Figure 6:**
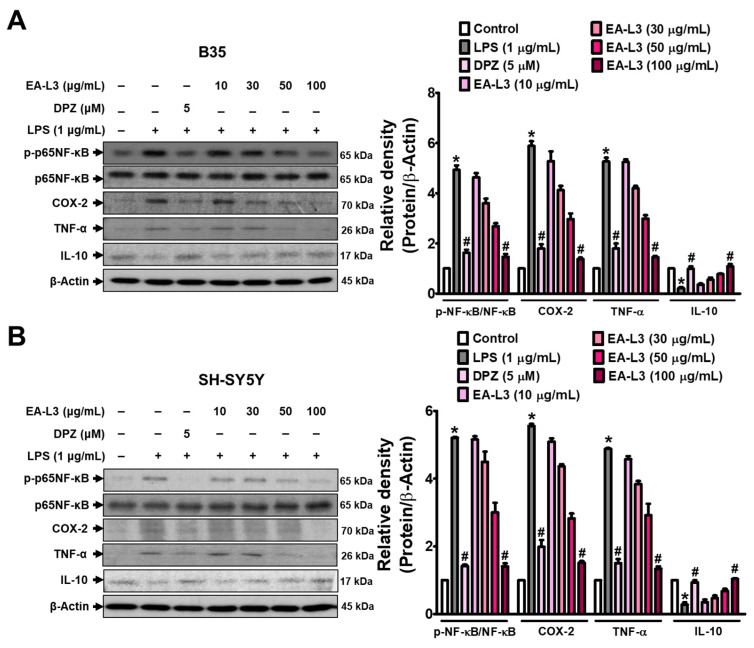
EA-L3 prevents LPS-induced expression of TNF-α and COX-2 while increasing the expression of IL-10 in neuroblastoma cell lines. The cell lines were treated alone or co-treated with LPS (1 μg/mL) and EA-L3 (10, 30, 50, and 100 μg/mL) or DPZ (5 μΜ) for 24 h, and total protein was extracted. Western blot was performed for the analysis of the protein levels of p-p65NF-κB, COX-2, TNF-α, and IL-10. Cell lines used were (**A**) B35 and (**B**) SH-SY5Y cells. The data presented are the means ± SD of three independent experiments and were analyzed by one-way ANOVA with Tukey’s post hoc test. * *p* < 0.05 vs. control group; # *p* < 0.05 vs. LPS-treated group.

Next, we verified the effect of EA-L3 on inflammatory markers in LPS-induced B35 and SH-SY5Y cells (Figure 6A,B, Figures S4 and S5). The respective cells were stimulated with LPS (1 μg/mL) with or without DPZ (5 μΜ) or EA-L3 at the indicated concentrations (10, 30, 50, and 100 μg/mL). The Western blots of 1 h of LPS stimulation in both neuroblastoma cells exhibited increased TNF-α protein expression compared with the vehicle-treated control. The pre-treatment of EA-L3 1 h before LPS stimulation subsided the TNF-α and COX-2 protein expression. At a dose of 100 μg/mL, EA-L3 showed the greatest reduction in the protein levels of TNF-α and COX-2 compared to those of the LPS-challenged cells. LPS-increased p65NF-κB phosphorylation also decreased with EA-L3 in neuroblastoma cells, as in BV2 microglia. The highest dose of EA-L3 (100 μg/mL) showed the strongest increase in IL-10 protein expression, similar to the DPZ-treated group. All these findings highlight that EA-L3 can regulate the neuroinflammatory process by achieving a balance between pro- and anti-inflammatory markers such as TNF-α, COX-2, and IL-10.

### 3.6. Effect of EA-L3 on Age-Induced Passive Avoidance in Mice

**Figure 7:**
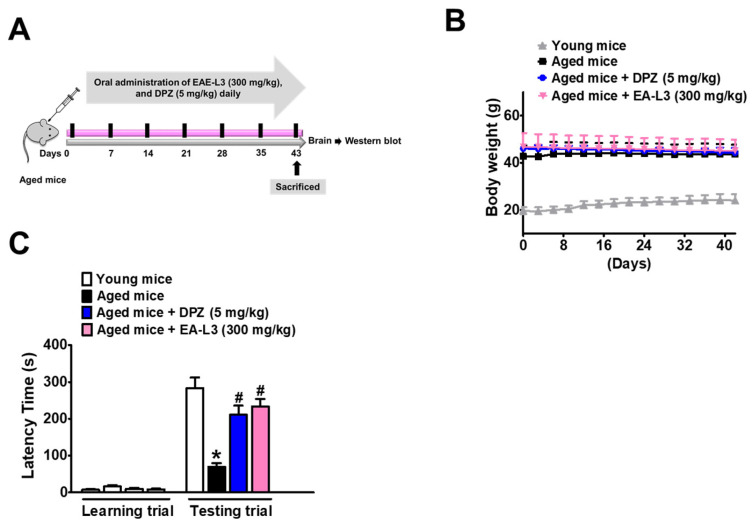
Oral administration of EA-L3 to aged mice results in significantly improved cognitive performance. (**A**) Experimental outline of aged mice. Aged mice were given oral administration of either normal saline, DPZ (5 mg/kg), or EA-L3 (300 mg/kg), respectively, for 42 days. (**B**) Body weight and (**C**) Passive Avoidance Test. *n* = 5 for 6-week mice groups and for 24-month mice groups. Data were shown as mean ± SD (*n* = 5). Significance was evaluated using a one-way ANOVA with Tukey’s post hoc test. * *p* < 0.01, compared with young mice; # *p* < 0.01, compared with vehicle-treated aged mice.

Figure 7A summarizes the results of EA-L3 therapy on the learning ability of aged mice. Aged mice and the drug-treated group showed no significant difference in body weight during the experimental time period (Figure 7B). The aged mice group, compared to the young mice group, required significantly longer times on the first day to enter the dark chamber, possibly because they were less mobile and less curious than their younger counterparts (Figure 7C). On the second day, aged control mice entered the dark room significantly faster than young control mice, indicating that they did not remember the electric shock. After 6 weeks of treatment with EA-L3 extract and DPZ, mice in the EA-L3 (300 mg/kg) and DPZ (5 mg/kg) groups displayed noticeably longer latency. Aged mice fed with EA-L3 and DPZ behaved very similarly to young control mice because they entered the dark chamber significantly faster than aged control mice on the first day and stayed in the light chamber significantly longer than aged control mice on the second day.

### 3.7. Effect of EA-L3 in Aged-Induced Neuroinflammation and Neurogenesis in C57BL/6 Mice

**Figure 8:**
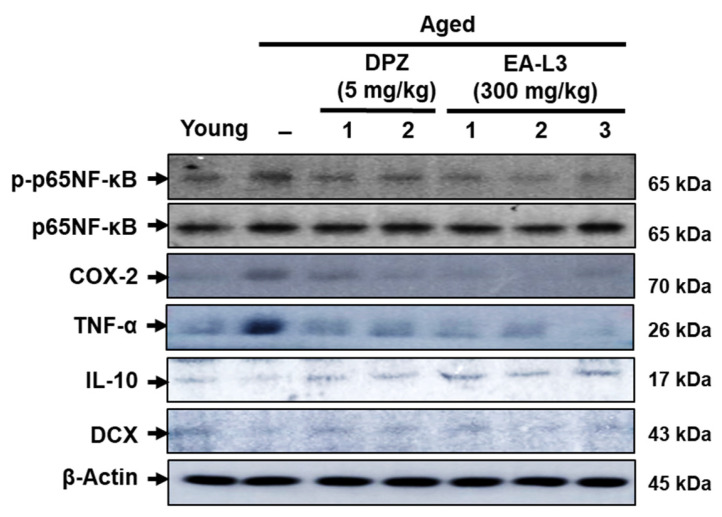
Effect of EA-L3 on the response of inflammatory and neurogenesis markers in aged mice. The concentration of p-p65NF-κB, COX-2, TNF-α, IL-10, and DCX in the hippocampus of young and aged mice was detected by Western blot and β-actin as a loading control. Numbers 1, 2, and 3 represent mice 1, 2, and 3.

The levels of TNF-α, IL-10, and COX-2 were measured in the brains of aged and EA-L3-administered mice. Aged mice had higher levels of proinflammatory mediators, TNF-α, and COX-2 protein expression, and a reduced expression of the anti-inflammatory cytokine IL-10. Administrations of EA-L3 extract decreased TNF-α and COX-2 protein expression in the hippocampus compared with untreated aged mice. In addition, EA-L3-treated mice showed increased IL-10 expression compared with untreated aged mice. EA-L3 supplementation also increased the neurogenesis marker DCX in aged mice (Figure 8). These results suggest that EA-L3 can reduce proinflammatory markers and increase anti-inflammatory and neurodevelopmental markers in aged mice.

### 3.8. Effect of EA-L3 in Aged-Induced Cognitive Impairment in C57BL/6 Mice

**Figure 9:**
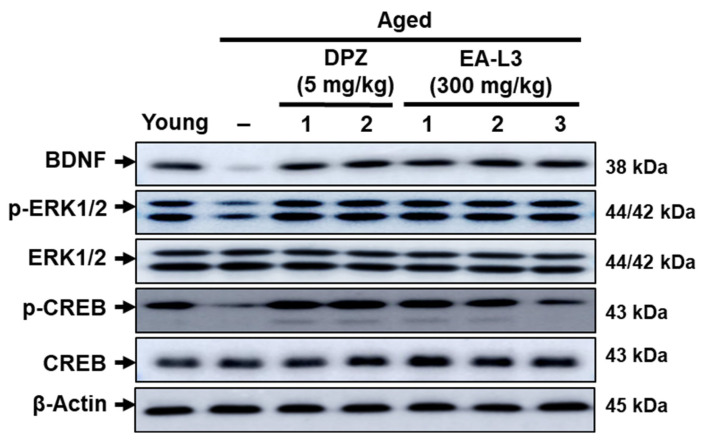
Representative Western blots of BDNF, p-ERK, and p-CREB in the hippocampus of young and drug-treated or untreated aged mice. The protein levels of BDNF, p-ERK, and p-CREB were normalized to β-actin expression. Numbers 1, 2, and 3 represent mice 1, 2, and 3.

By using Western blot analysis, we were able to examine the expression levels of BDNF, p-ERK, and p-CREB in order to gain insight into the potential mechanism of cognitive enhancements in the brain tissue of elderly mice for EA-L3. As the hippocampus is central to learning and cognitive functions in the brain, BDNF expression was found to decrease in the hippocampus of aged mice. However, EA-L3 (300 mg/kg) treatment regained hippocampal BDNF protein expression in aged mice compared with vehicle-treated aged mice. The recovery of BDNF by EA-L3 treatment was consistent with DPZ treatment (Figure 9). In addition, because the BDNF-induced activation of the ERK/CREB signaling pathway is crucial for hippocampal neurogenesis and cognition improvement, we also examined the related phosphorylation of ERK and CREB. The administration of EA-L3 and DPZ increased ERK and CREB phosphorylation in the hippocampus of aged mice, similar to that of young mice. These results indicate that EA-L3 can improve age-dependent hippocampal cognitive decline by activating ERK/CREB signaling and increasing BDNF protein expression.

## 4. Discussion

The aging process is marked by chronic, low-grade systemic inflammation in the absence of overt infection, constituting a significant risk factor for morbidity and mortality [37]. Brain aging is defined by cognitive decline and memory deficits, where the major mechanisms involved are oxidative stress and neuroinflammation [38]. Phospholipids in the brain are sensitive to ROS-induced damage [39]. Therefore, the consumption of plant extracts rich in antioxidants such as flavonoids, phenolic acids, stilbenes, lignans, and tannins is known to prevent age-related memory decline in middle-aged mice as they modulate cell and molecular processes during learning and memory functioning. It is worth noting that the leaves of EA extract are rich in polyphenols like quercetin and rutin, which help enhance memory function [32,40].

Our previous study showed that supplementation with EA twig extract improved cognitive impairment in a scopolamine-induced mouse model [31]. The work presented here provides EA leaf extracts and their potential anti-aging effects on oxidative stress, cognitive decline, and neuroinflammation in vitro and in vivo models. In the current investigation, EA-L1, EA-L2, and EA-L3—three distinct EA leaf extracts—were formulated. EA-L3 was found to be less cytotoxic to RAW264.7, BV2, B35, and SH-SY5Y cells than the other two extracts, EA-L1 and EA-L2. Additionally, EA-L3 showed minimal cytotoxicity in neuroblastoma and macrophage cell lines, even at concentrations higher than 500 μg/mL. The HPLC analysis of EA-L1 demonstrated the presence of compounds like pheophytin c1 and pheophorbide a and b. EA-L3 revealed no such compounds, which may account for its less cytotoxic effect. Research has indicated that the presence of chlorophyll-related compounds may affect the natural product’s analytical qualities [41]. Thus, the EA-L3 extract was selected for additional testing in aged and neuroblastoma models.

Numerous flavonoids, such as catechin, quercetin, rutin, kaemferol, and triterpenes, have been shown to be able to pass across the blood–brain barrier (BBB) and potentially reach the central nervous system. This ability is associated with some neuroprotective benefits that have already been reported for this class of flavonoids in the literature [42,43,44]. Therefore, we checked the in vitro effects of EA-L3 by using neuroblastoma and microglial cells. In our study, the chemical characterization of the EA-L3 extract by HPLC/MS analysis allowed for the identification of three polyphenolic compounds like quercetin, rutin, and kaemferol, as well as a triterpene, abruslactone A. Studies have shown that kaemferol and its derivatives reduce cholinesterase activity, prevent microglial activation, remove amyloid fibrils, and safeguard the blood–brain barrier [45]. In LPS-stimulated RAW264.7 cells and BV2 microglia cells, triterpenes (abruslactone A and demethylregelin), caffeic acid, and chlorogenic acid extracted from EA leaves and twigs are known to contribute to anti-inflammatory effects [29,46]. In addition, it was discovered that the anti-inflammatory properties of quercetin and kaemferol in EA also inhibited inflammatory mediators, including COX-2 and nitric oxide synthase [47]. It was also shown that neolignans extracted from EA leaf and twig extracts contributed to the anti-inflammatory benefits by inhibiting prostaglandin E2 and inflammatory cytokines. [32,48].

Behavioral studies in the mouse model showed that cognitive domains such as memory and learning were particularly impaired in aged mice and suggested that aged mice are more vulnerable to memory impairments than young mice. We investigated the safety of a single oral injection of EA-L3 in ICR mice. EA-L3 was administered at all doses without affecting body weight. Although no abnormal clinical signs and behaviors were recorded, one mouse treated with 1000 mg/kg of EA-L3 died. All the organs investigated in the mouse autopsy revealed no gross pathological findings and no significant differences in the liver, lungs, kidneys, heart, stomach, or spleen, as compared to the control group. According to these findings, 300 mg/kg or less of a single oral dose of EA-L3 was considered a safe dose for ICR mice (Figure S1). Therefore, the PAT demonstrated that supplementing old animals with EA-L3 extracts (300 mg/kg) significantly improved cognitive impairment as compared to young mice. In support of our behavioral test, we also found that EA-L3 increased the age-related reductions in BDNF/p-ERK/p-CREB expressions in the hippocampus. Consequently, EA-L3 treatment had a similar effect on BDNF, ERK, and CREB in scopolamine-induced B35 and SH-SY5Y cells. The treatment with EA-L3 increased the protein level of BDNF, p-ERK, and p-CREB compared to the vehicle-treated control. The accumulated evidence has shown that ERK is a signaling molecule downstream of BDNF and that ERK phosphorylation activates CREB transcription factor proteins that regulate memory formation and synaptic remodeling. These results suggest that the involvement of BDNF/p-ERK/p-CREB signaling occurs during the cognition-enhancing effects of EA-L3, generating new brain cells and strengthening existing ones to avert the consequences of aging. It has also been demonstrated that BDNF can interact with oxygen radicals (ROS), an imbalance associated with aging processes [38]. On the other hand, BDNF also increases CREB phosphorylation and its nuclear localization by reducing the binding of NF-κB and CBP, which further inhibits the production of proinflammatory cytokines. Therefore, BDNF/TrkB signaling is crucial for controlling microglial activation [36]. However, further studies need to be performed to confirm this relationship in our study.

Declining antioxidant levels and increased lipid peroxidation with age are hallmarks of aging. Studies have shown that increased oxidative stress during aging leads to inflammation [42]. Various investigations have shown that LPS is present in the aged brain with an increased concentration in surrounding and affected neurons in AD [49]. It is assumed that LPS produced by gut microbes would be able to pass through the blood–brain barrier (BBB) and the gastrointestinal tract to enter the systemic circulation and the brain [50]. The current study demonstrates that treating EA-L3 in LPS-induced microglial cells dramatically lowers ROS levels. Microglial cells were chosen as they constitute the first line of immune response in the brain. Furthermore, the NF-κB pathway is a significant nuclear transcription factor involved in inflammation that can react to signals like oxidative stress. When this system is activated, TNF-α and IL-6 production rise [43]. Therefore, our findings indicate that EA-L3 inhibited NF-κBp65 phosphorylation in the hippocampus and microglia of the aged mice brains, indicating a decrease in the transcription of genes such as TNF-α and COX-2, regulated by these transcription factors. The findings observed in aged mice also mirror those obtained in LPS-induced neuroblastoma and microglia cell lines compared with untreated controls. Many studies have shown that neurons are able to release inflammatory mediators like IL-6, IL-1, etc., [51]. In neuroblastoma SH-SY5Y cells, previous studies observed that LPS treatment resulted in the enhanced synthesis of proinflammatory cytokines like TNF-α, IL-1β, and IL-6 [52], while there was a decrease in anti-inflammatory cytokines like IL-10 [53]. Therefore, both neuroblastoma cells and macrophages stimulated by LPS were employed as models for evaluating anti-inflammatory activities of the EA-L3 extract. More interestingly, EA-L3 diminished the expressions of TNF-α and COX-2 after LPS induction in BV2, B35, and SH-SY5Y as well as in the hippocampus of aged mice. Our findings are consistent with earlier research, which found that injecting LPS peripherally into an aged mouse model increased and sustained the neuroinflammatory response by producing high levels of TNF-α and IL-1β in the hippocampus [54]. Further, these activated microglia-produced proinflammatory cytokines also induce a chronic inflammatory response and lead to neuronal damage [18].

In contrast, EA-L3 treatment also increased the expression of M2 markers such as IL-10 in aged mice. Our findings are consistent with studies conducted on transgenic mice, where it has been demonstrated that IL-10 reduces neuroinflammation by promoting neurogenesis and boosting cognitive abilities [55]. Coronal sections of the brains of the aged animals show higher levels of IL-6 secretion, but lower compared with adult brains [42]. Cytokines like IL-4, IL-10, IL-13, and transforming growth factor-β (TGF-β) are involved in microglia neuroprotection and tissue healing [56,57,58]. We can assume that the EA-L3 extract’s anti-inflammatory actions and effects on cognitive enhancement resulted from an M1 phenotype transition to the M2 phenotype in the aged mice’s brains.

Along with the inflammatory markers, increased DCX expression was also observed in the hippocampus of the aged mice. Neurons express the cytosolic protein DCX, which is frequently used as a diagnostic tool for neurogenesis [59]. Aging leads to a decrease in the amount of DCX in the DG and damages the dendritic growth of the immature new neurons [60]. Importantly, this reduction in DCX expression was attenuated by the administration of the EA-L3 extract in mice, indicating that EA-L3 promotes the growth of immature neurons in the mice’s hippocampi. In addition, our data is in line with the knowledge that inflammatory cytokines such as TNF-α via Rho kinase can repress neurite development and branching during inflammation [61].

Altogether, we investigated the anti-cognitive and anti-neuroinflammatory nature of EA-L3 extract. Cognitive decline in aged mice may be associated with increased cytokines and oxidative stress. EA-L3 inhibitions on ROS, NF-κBp65 phosphorylation, and subsequent TNF-α and COX-2 proteins may be involved in reducing age-related cognitive deficits. The biological alterations that occurred simultaneously may have been ascribed to the protective properties of triterpenes and flavonoids included in EA leaf extracts, although the presence of substances containing chlorophyll interfered with these qualities in EA-L1. Furthermore, the role of increased BDNF in aged mice treated with EA-L3 has not been fully characterized, and its mechanism of preventing neuroinflammation needs further investigation.

## 5. Conclusions

As a result, the current study suggests that aging-related chronic inflammation impairs memory-related molecules (BDNF/ERK/CREB) by raising ROS and pro-inflammatory mediator levels such as NF-κB, TNF-α, and COX-2 and lowering IL-10 levels in the hippocampus. These cognitive abnormalities were reversed by EA-L3 extract in aged mice and in scopolamine-induced neuroblastoma cells. EA-L3 extract also showed similar patterns in LPS-induced neuroblastoma and microglia cells. Based on these findings, we were able to show that the antioxidant and anti-inflammatory effects of EA-L3 are protective and may improve cognitive function during normal aging. The treatment also enhanced neurogenesis, indicating the potential therapeutic value of EA-L3 in aging and aging-related conditions.

## Data Availability

Data are contained within the article and Supplementary Materials.

## Supplementary Materials

The following supporting information can be downloaded at: https://www.mdpi.com/article/10.3390/antiox13040433/s1, Figure S1: Single dose toxicity test of EA-L3; Figure S2: Set 2 and 3 for Figure 4A,B in main text; Figure S3: Set 2 and 3 for Figure 5B in main text; Figure S4: Set 2 and 3 for Figure 6A in main text; Figure S5: Set 2 and 3 for Figure 6B in main text.
