# Supplementary material for: Euonymus alatus Leaf Extract Attenuates Effects of Aging on Oxidative Stress, Neuroinflammation, and Cognitive Impairment
Source: Antioxidants (Basel). 2024 Apr 2;13(4):433. doi: 10.3390/antiox13040433 (PMC11047375; doi:10.3390/antiox13040433)
Supplement: Supplementary file 1 [file antioxidants-13-00433-s001.zip › antioxidants-2850743-supplementary.pdf]

# Supplementary Files

## *Euonymus alatus* Leaf Extract Attenuates Effects of Aging on Oxidative Stress, Neuroinflammation, and Cognitive Impairment

**Pallavi Gurung, Junmo Lim, Til Bahadur Thapa Magar, Rajeev Shrestha and Yong-Wan Kim ***

Dongsung Cancer Center, Dongsung Pharmaceuticals Corporation, Daegu 41061, Republic of Korea
*Correspondence: kyw17@ds-pharm.co.kr

**TABLE OF CONTENTS**

**S1. Single drug toxicity test of EA-L3**

**S2. Set 2 and 3 for Fig 4A and 4B in main text.**

**S3. Set 2 and 3 for Fig 5B in main text.**

**S4. Set 2 and 3 for Fig 6A in main text.**

**S5. Set 2 and 3 for Fig 6B in main text.**

## S1. Single drug toxicity test of EA-L3

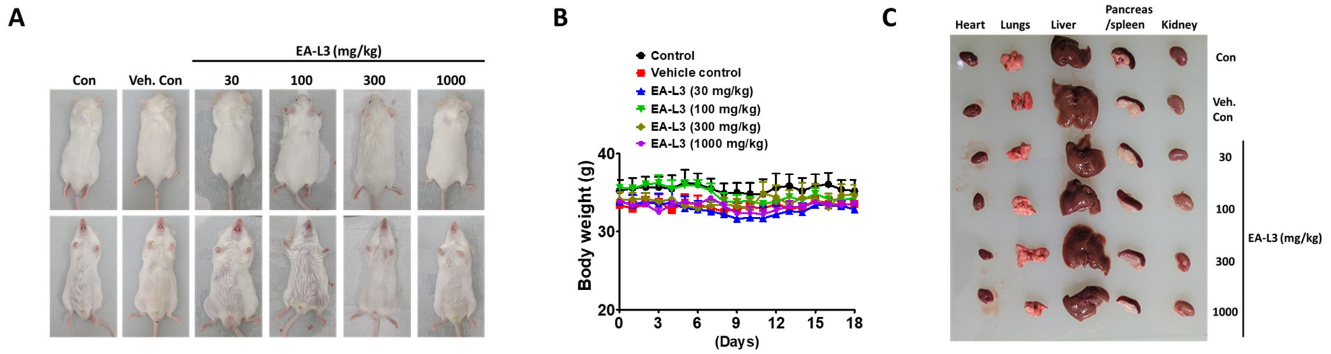


**Figure S1. A single dose toxicity test of the mice administered with EA-L3 for 18 days.** 18 (five-week-old male) ICR mice of similar bodyweights were chosen and assigned to one of 6 groups: negative control; vehicle control; 30 mg/kg ; 100 mg/kg; 300 mg/kg and 1000 mg/kg of EA-L3 respectively. EA-L3 was administered by an oral injection to evaluate toxicity to the drug. The vehicle control groups received the same volume of vehicle (normal saline), respectively. All abnormal clinical signs and behaviors were recorded, immediately 1, 2, and 3 h after drug or vehicle administration, and every morning. A. Photographs of representative mice in each group B. Mean body weight C. Representative images of the mice vital organs.

## S2. Set 2 and 3 for Fig 4A and 4B in main text.

### Set 3

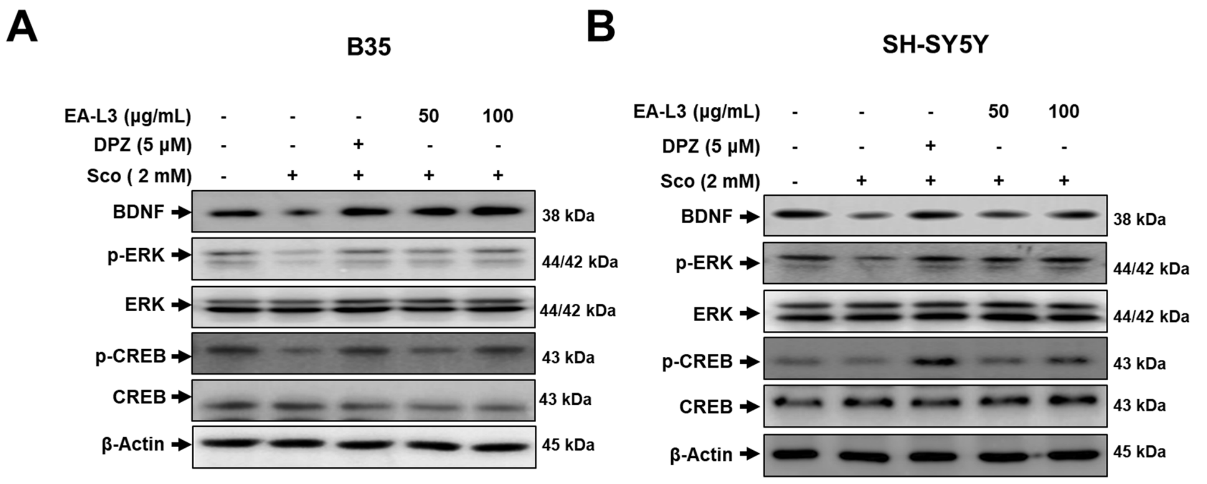


**Figure S2**: Effect of EA-L3 on scopolamine-triggered expression of memory-related molecular markers in B35 and SH-SY5Y cells. Cells were treated with EA-L3 at the indicated concentration, and the extracted protein was analyzed to

determine BDNF, p-ERK, and p-CREB protein levels. Set 2 and Set 3 of Figure 4 (**A**) B35 and (**B**) SH-SY5Y cells in main text.

## S3. Set 2 and 3 for Fig 5B in main text.

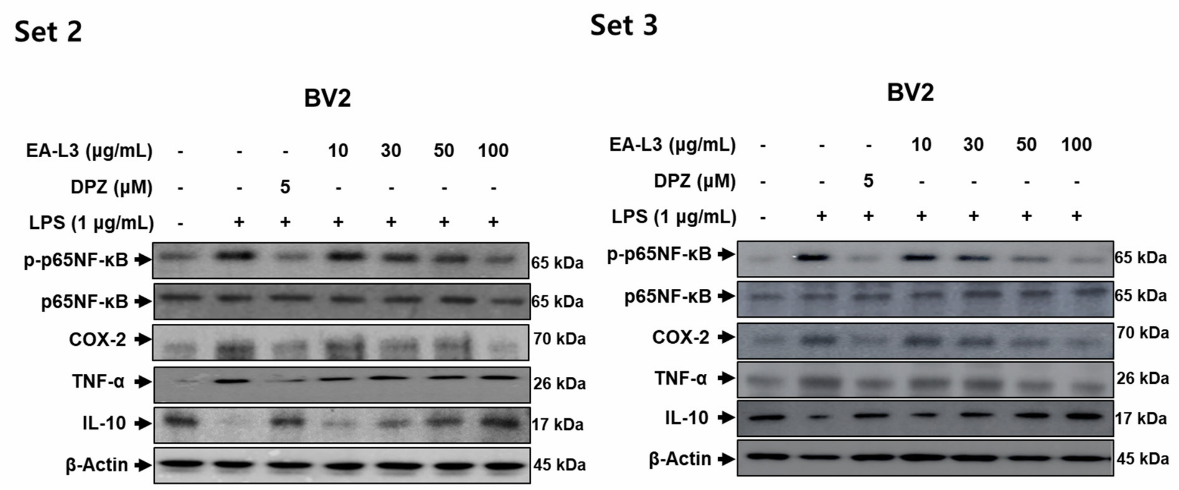


**Figure S3**: Effect of EA-L3 on LPS-induced inflammatory markers in BV2 microglial cells. The cell lines were treated alone or co-treated with LPS (1 μg/mL) and DPZ (5 μM) or EA-L3 (10–100 μg/mL) for 24 h and total protein was extracted. A Western blot was performed for the analysis of p-p65NF-κB, COX-2, TNF-α, and IL-10 protein levels, respectively. Set 2 and Set 3 for Fig 5B in main text.

## S4. Set 2 and 3 for Fig 6A in main text.

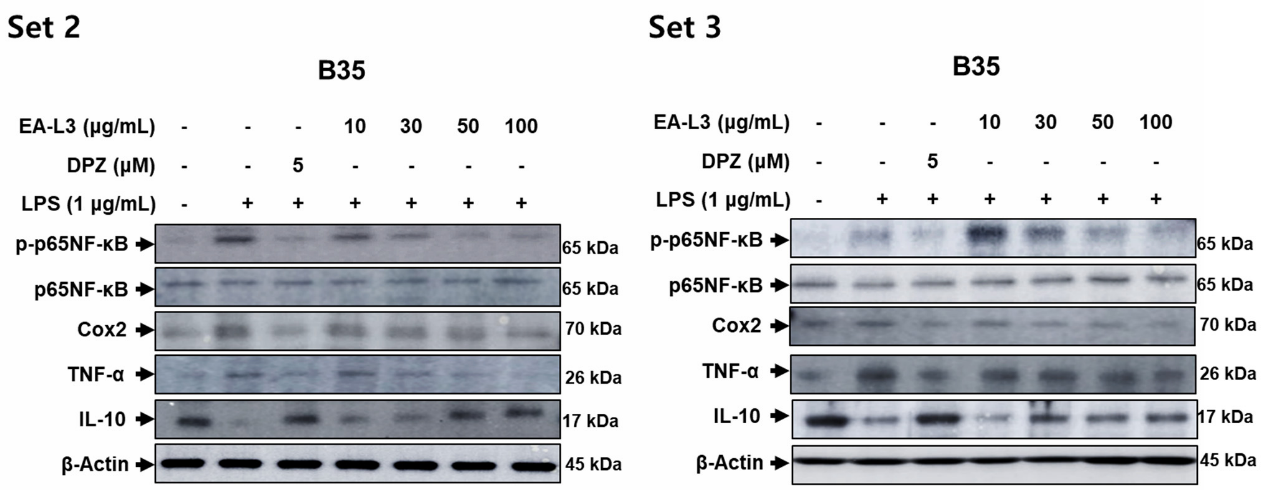

**Figure S4**: Effect of EA-L3 on LPS-induced inflammatory markers in B35 cells. The cell lines were treated alone or co-treated with LPS (1 μg/mL) and DPZ (5 μM) or EA-L3 (10–100 μg/mL) for 24 h and total protein was extracted. A Western blot was performed for the analysis of p-p65NF-κB, COX-2, TNF-α, and IL-10 protein levels, respectively. Set 2 and Set 3 for Fig 6A in main text.

## S5. Set 2 and 3 for Fig 6B in main text.

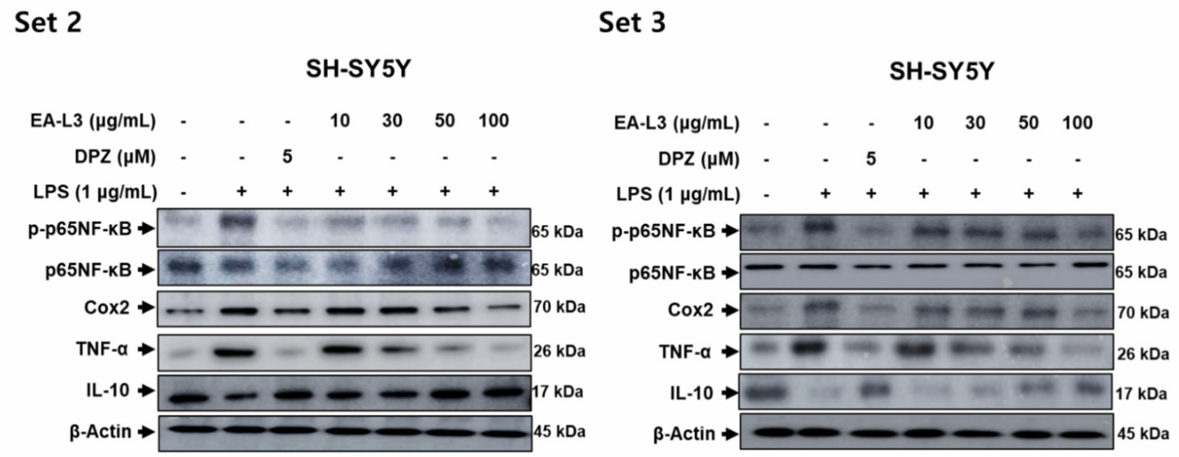


**Figure S5**: Effect of EA-L3 on LPS-induced inflammatory markers in SH-SY5Y cells. The cell lines were treated alone or co-treated with LPS (1 μg/mL) and DPZ (5 μM) or EA-L3 (10–100 μg/mL) for 24 h and total protein was extracted. A Western blot was performed for the analysis of p-p65NF-κB, COX-2, TNF-α, and IL-10 protein levels, respectively. Set 2 and Set 3 for Fig 6B in main text.
